# A simple in vitro biomimetic perfusion system for mechanotransduction study

**DOI:** 10.1080/14686996.2020.1808432

**Published:** 2020-09-11

**Authors:** Ruikang Xue, Sarah Cartmell

**Affiliations:** Department of Materials, School of Natural Sciences, Faculty of Science and Engineering, University of Manchester, Manchester, UK

**Keywords:** Mechanotransduction, flow-induced shear stress, biomimetic perfusion system, mesenchymal stem cell, in vitro, 211 Scaffold / Tissue engineering/Drug delivery

## Abstract

In mechanotransduction studies, flow-induced shear stress (FSS) is often applied to two-dimensional (2D) cultured cells with a parallel-plate flow chamber (PPFC) due to its simple FSS estimation. However, cells behave differently under FSS inside a 3D scaffold (e.g. 10 mPa FSS was shown to induce osteogenesis of human mesenchymal stem cells (hMSC) in 3D but over 900 mPa was needed for 2D culture). Here, a simple in vitro biomimetic perfusion system using borosilicate glass capillary tubes has been developed to study the cellular behaviour under low-level FSS that mimics 3D culture. It has been shown that, compared to cells in the PPFC, hMSC in the capillary tubes had upregulated Runx-2 expression and osteogenic cytoskeleton actin network under 10 mPa FSS for 24 h. Also, an image analysis method based on Haralick texture measurement has been used to identify osteogenic actin network. The biomimetic perfusion system can be a valuable tool to study mechanotransduction in 3D for more clinical relevant tissue-engineering applications.

## Graphical abstract

**Figure:**
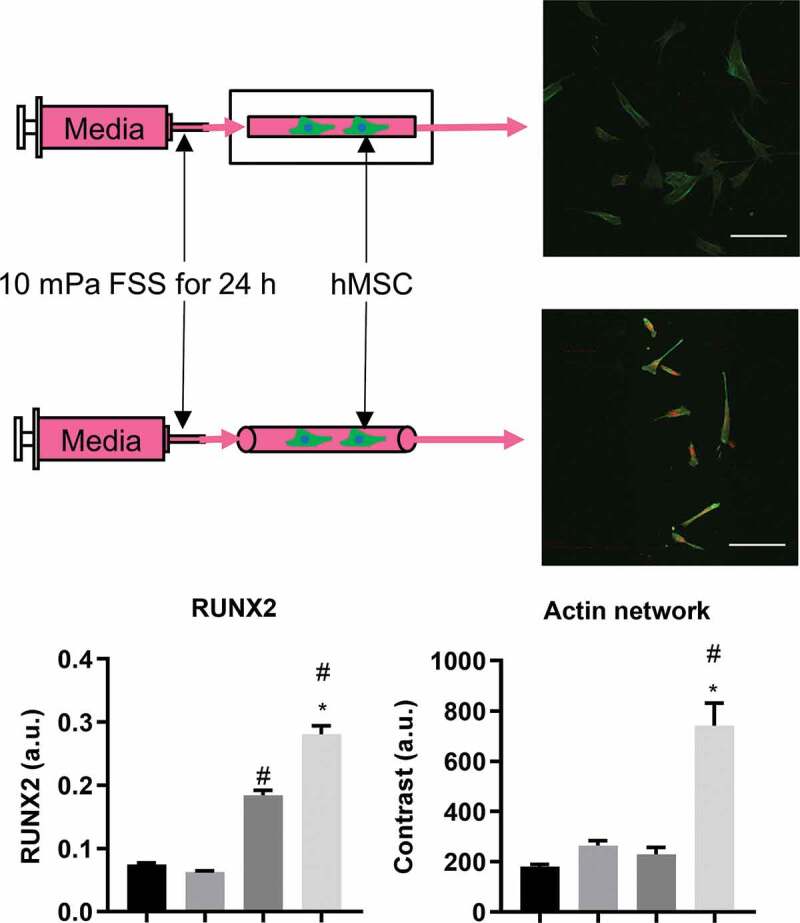

## 1. Introduction

Mechanotransduction studies of 2-dimensional (2D) cultured cells widely employ parallel-plate flow chambers (PPFC) to generate readily calculated flow-induced shear stress (FSS) [1,2]. Mechanotransduction studies of cells inside a 3D porous scaffold with a perfusion bioreactor are more physiologically relevant. However, it often needs complex mathematical modelling such as computational fluid dynamics (CFD) to estimate the FSS [3].

It was reported that low-level FSS (1 to 10 mPa) promoted osteogenic differentiation of cells inside a 3D scaffold whilst significantly higher FSS (100 to 4000 mPa) was required for osteogenesis in 2D culture [4–8]. Compared to 2D culture, 3D system provides additional geometric cues to cells. Ferlin et al. showed that, during 3D culture, the scaffolds with cylindrical pores led to early human mesenchymal stem cells (hMSC) osteogenesis whereas those with cubic pore induced chondrogenesis and adipogenesis [9].

In this study, we hypothesized that the curved surface from a porous scaffold can change cellular behaviour in vitro. Thus, we developed a simple biomimetic perfusion system using borosilicate glass capillary tubes to study the behaviour of cells cultured on curved surface and their response to low-level FSS in vitro. The capillary tube has a diameter of 560 µm, mimicking the pore size of human trabecular bone [10]. Unlike 3D culture, the FSS in the system can be readily calculated. hMSC were cultured in the capillary tubes and in the PPFC under static condition or 10 mPa FSS for 24 h. Their Runt-related transcription factor-2 (Runx-2) and actin network were investigated. To distinguish different actin networks, Haralick texture measurement which quantifies correlations between pixels at a specific distance was employed [11].

## 2. Materials and Methods

### 2.1. Biomimetic perfusion system

**Figure 1.**
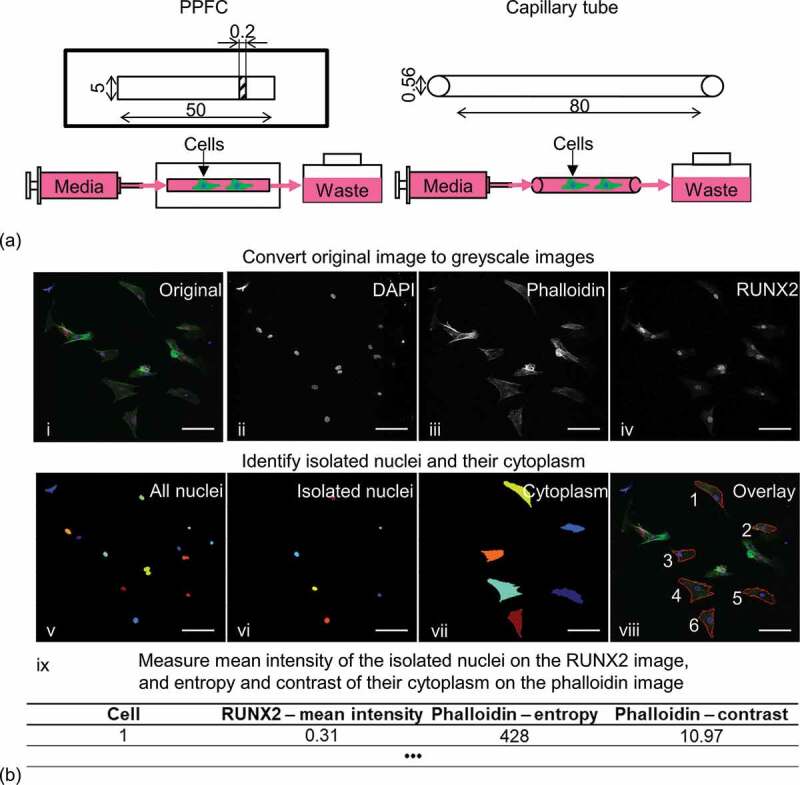
(a) Schematic of the parallel-plate flow chamber (PPFC) and the biomimetic perfusion system using a borosilicate glass capillary tube. Dimensions are in mm. (b) Image analysis steps to quantify actin network and Runx-2 expression of isolated cells. Scale bar: 100 µm.

Cylindrical capillary tubes (Marienfeld) with 80 mm length and 560 µm inner diameter were used in the biomimetic perfusion system (Figure 1(a)). They had biocompatible borosilicate glass surface for cell culture and uniform curvature which is controlled by the tube diameter. When connected to a syringe pump, the FSS inside the capillary tube can be calculated through Equation 1 [12].

$$\tau =\frac{32Q\mu }{\pi d^{3}}$$

Equation 1. Calculation of FSS in the biomimetic perfusion system, where τ is the FSS (Pa), µ is the dynamic viscosity (Pa∙s), Q is the flow rate (m^3^/s), d is the tube inner diameter (m).

PPFC with tissue culture plastic surface [12] with 50 mm channel length, 5 mm width and 200 µm height were included for comparison. The FSS inside the PPFC can be calculated through Equation 2 [13].

$$\tau =\frac{32Q\mu }{\pi d^{3}}$$

Equation 2. Calculation of FSS in the PPFC, where τ is the FSS (Pa), Q is the flow rate (m^3^/s), µ is the dynamic viscosity (Pa∙s), w is the channel width (m) and h is the channel height (m).

### 2.2. Cell culture and experimental set-up

hMSC (Lonza) in passage 4–6 from two different donors were used in the study. Cells were cultured in the growth medium – Dulbecco’s Modified Eagle Medium (Sigma-Aldrich) supplemented with 10% foetal bovine serum (Sigma-Aldrich) and 1% Antibiotic Antimycotic Solution (Sigma-Aldrich). Cells were trypsinised and 50,000 cell/ml cell suspension in the growth medium was prepared. A droplet of 200 µl cell suspension was placed in the centre of a petri dish. One end of the capillary tube was inserted into the droplet and cell suspension was drawn into the tube due to capillary force. For the PPFC, 100 µl cell suspension was added to the channel. Before perfusion, cells were incubated in the growth medium at 37°C 5% CO_2_ for 24 h for attachment.

To set up the perfusion system, the capillary tubes or PPFC were connected to a syringe pump (Chemyx) and a 1.5 ml Eppendorf tube as waste container through 0.5 mm inner diameter tubing ([13], Figure 1(a)). A FSS of 10 mPa was applied to the cells through growth medium perfusion in the capillary tubes and PPFC at the respective 10 and 20 μl/min flow rates for 24 h at 37°C 5% CO_2_. Static controls were also included in the experiment. All samples were composed of three independent samples from two donors (n = 6).

### 2.3. Fluorescence staining and imaging analysis

Immediately after perfusion, cells inside the capillary tubes and the PPFC were fixed, permeabilised and blocked. They were then incubated with the conjugated rabbit anti-RUNX2 647 antibody (ABCAM) at 1:200 dilution at 4°C overnight. Afterwards, they were incubated with DAPI (Thermofisher Scientific) at 1:1000 dilution and Alexa Fluor 488 phalloidin (Thermofisher Scientific) methanol stock solution at 1:40 dilution at room temperature for 1 h.

Fluorescence images were captured with a Leica SP8 confocal microscope equipped with a 10× objective (Leica Microsystems). DAPI, phalloidin and Runx-2 data were captured using the line sequential scanning mode with their respective fluorescence spectrum. Laser power and detector settings were kept constant across samples. For each image, a minimum of 20 z-stacks were recorded at 5 µm z-spacing at 1024 × 1024 pixels. A projection image was generated from each image stack through maximum intensity algorithm.

The projection images were loaded into CellProfiler [14] for semi-quantitative analysis of nuclear Runx-2 expression and action network. The original data were firstly split into three greyscale images of the respective DAPI, phalloidin and Runx-2 stains. Then, the nuclei from the isolated cells were identified through the DAPI image followed by manually removing overlapping and bordered cells. Afterwards, the cytoplasm related to the nuclei was identified through the phalloidin image. The fluorescence intensity of the nuclei was then measured using the Runx-2 image. Lastly, two Haralick texture descriptors, contrast and entropy, were measured at the identified cytoplasm using the phalloidin image (Figure 1(b)). In brief, contrast was a measure of local variation in an image and entropy was a measure of the complexity within an image [11].

One-way ANOVA with Tukey post hoc test was conducted for statistical analysis, where P smaller than 0.05 was considered as statistically significant.

## 3. Results

**Figure 2.**
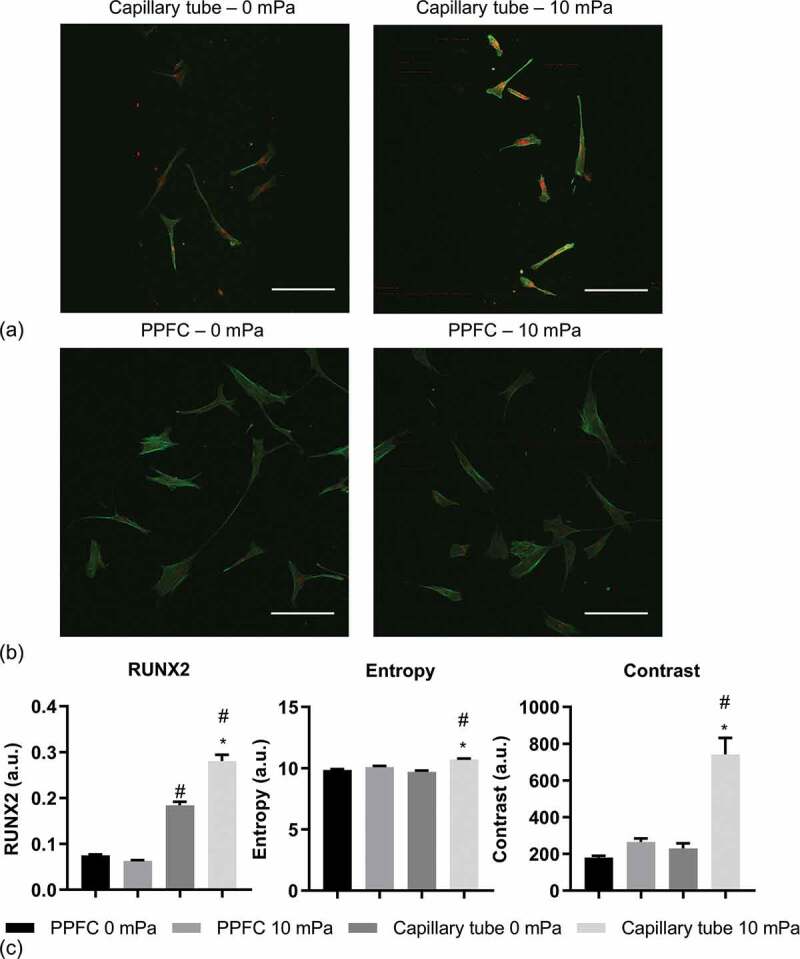
Representative projection images of hMSC under static (0 mPa) or 10 mPa FSS in capillary tubes (a) and PPFC (b). Runx-2 is in red, phalloidin is in green, and DAPI (blue) has been removed for better visualisation. Scale bar: 250 µm. (c) Quantification of Runx-2 and action texture entropy and contrast. #: significant difference (P < 0.05) between the PPFC and capillary tubes. *: significant difference between the 10 mPa and 0 mPa samples. n = 6.

Figure 2(a and b) show representative projection images with Runx-2 in red and phalloidin in green of hMSC under static (0 mPa) or 10 mPa FSS in the respective capillary tube and PPFC; the blue channel (DAPI) has been removed for better visualisation. In terms of Runx-2 expression, compared to the PPFC, hMSC had increased Runx-2 expression in the capillary tubes both under static and perfusion conditions. Quantitative analysis revealed that, in the PPFC, 10 mPa FSS led to no significant change in Runx-2 expression (Figure 1(c)). In contrast, perfusion led to significant upregulation of Runx-2 of the cells in the capillary tubes.

Qualitatively, Figure 2(a and b) showed that actin network of hMSC in the capillary tube under 10 mPa had increased stress fibre formation with higher fluorescence intensity and reduced homogeneity across individual cells compared to other conditions. The quantification of texture confirmed the observation (Figure 2(c)). It showed that, under static culture, cells in the PPFC and the capillary tube had comparable entropy and contrast; in contrast, under perfusion, the cells in the capillary tube had significantly higher entropy and contrast than those in the PPFC.

## 4. Discussion

Previously, demineralised freeze-dried human trabecular bone has been used as a tissue-engineering scaffold [10], so investigating the role of FSS through pores of this size as seen locally by MSCs in a direct perfusion system is relevant. Also, the FSS applied here was comparable to that seen in scaffold pores as determined via computer simulation when inlet flow rates into bioreactor were 10 µl/min which kept cells viable [4,15–17].

Runx-2 is considered by some investigators as the most important factor involved in osteogenesis and it has become a marker of early osteogenic differentiation [18]. Mechanical forces were shown to upregulate Runx-2 of hMSC as early as 24 hours after stimulation [19]. The cells in the PPFC were expected to have limited response to the applied low-level FSS since most literature indicated that significantly higher FSS was required to promote osteogenic differentiation of 2D cultured hMSC (reviewed in [8]). For example, increased osteogenesis was observed with 900 mPa FSS inside a PPFC [6]. Interestingly, Kim et al. reported that ~13 mPa FSS upregulated Runx-2 expression of 2D cultured hMSC inside a microfluidic device [20]. This discrepancy could originate from the synergistic effect of the use of osteogenic medium in combination with FSS.

During hMSC osteogenesis with differentiation medium, it was reported that hMSC actin network changed from a large number of thin, aligned filaments distributed across the cytoplasm to a few thick actin stress fibres found at the outermost periphery [21,22]. Moreover, actin modification was shown to happen as early as 24 hours [23]. It was reported that, under 1900 mPa FSS, the cytoskeletal actin network of cells cultured in PPFC had increased actin aggregation and stress fibre formation [1]. However, to the best of authors’ knowledge, there have been no studies on actin cytoskeleton of hMSC cultured under low-level FSS in the literature. The actin texture quantification results from this study showed higher entropy and contrast of hMSC in the capillary tubes under perfusion, indicating increased osteogenesis according to the literature.

Taking together, under 10 mPa FSS, hMSC in the capillary tube showed tendency towards osteogenesis through upregulated Runx-2 and osteogenic actin network, mimicking conditions observed inside a 3D porous scaffold [8]. Interestingly, increased Runx-2 expression was observed in the cells in the capillary tube even without perfusion, indicating the effect of the curved surface on cellular behaviour. Similarly, it was reported that ordered cylindrical pores inside a 3D printed scaffold led to significant upregulation of early osteogenic markers compared to cubic pores [9]. Werner et al. showed that hMSC were able to sense and differentially attach various concave and convex surface features created through stereolithography [24].

Apart from the difference in geometry between the capillary tubes and the PPFC, different flow rates were applied in the experiment in order to obtain the same FSS. Flow rate was reported to affect hMSC behaviour cultured in a β-tricalcium phosphate scaffold; under the same FSS, compared to the 3 ml/min flow rate, the 9 ml/min flow improved the secretion of osteopontin but depressed the expression of osteocalcin [5]. However, this study used significantly lower flow rates (10 and 20 µl/min) and their influence was expected to be minimum.

As an early-stage study with the biomimetic perfusion system, semi-quantitative image-based analysis was used here. Nevertheless, with strict control over fluorescence labelling and image acquisition, it was reported that quantitative immunofluorescence was able to obtain linear and reproducible results that were comparable to mass spectrometry [25]. Morphometric analysis of actin network was reported in several publications [26–28]. However, complicated algorithms with a large number of shape descriptors (e.g. filament number, length and orientation) were used, which increased the difficulty in data processing and interpretation. Here, Haralick texture measures were used to distinguish osteogenic actin network of hMSC; this method was further confirmed with alkaline phosphatase (ALP) expression of hMSC cultured in growth medium and osteogenic medium (Fig S1). Texture measures were successfully employed to distinguish a range of other phenotypes, which form the foundation of the majority of current automated image classification system [29].

In future, more mechanotransduction studies can be conducted using the biomimetic perfusion system to link the wealth of experience gained from 2D studies to more clinically relevant 3D culture. Also, the biomimetic perfusion system has potential to provide insights for vascular tissue engineering where flow inside a vessel plays a crucial role [30]. However, when cell suspension or blood-analogue is employed, non-Newtonian flow behaviour needs to be considered in FSS quantification [31].

## 5. Conclusions

In conclusion, we developed a simple biomimetic perfusion system using capillary tubes to study the cellular behaviour of hMSC on a curved surface under perfusion. An image analysis method based on Haralick texture measurement was applied to distinguish osteogenic actin network organisation. Results showed that hMSC in the capillary tubes and the PPFC had different cellular responses including actin network and Runx-2 expression to the applied low-level FSS. Under perfusion, hMSC in the capillary tube showed the behaviour similar to that described in 3D culture. With the biomimetic perfusion system, researchers will be able to study the cellular behaviour of cells similar to those in 3D environment and tissue-engineering settings.

## Supplementary Material

Supplemental Material
