# Supplementary figures and images for: A simple in vitro biomimetic perfusion system for mechanotransduction study
Source: Sci Technol Adv Mater. 2020 Sep 11;21(1):635–40. doi: 10.1080/14686996.2020.1808432 (PMC7534211; doi:10.1080/14686996.2020.1808432)

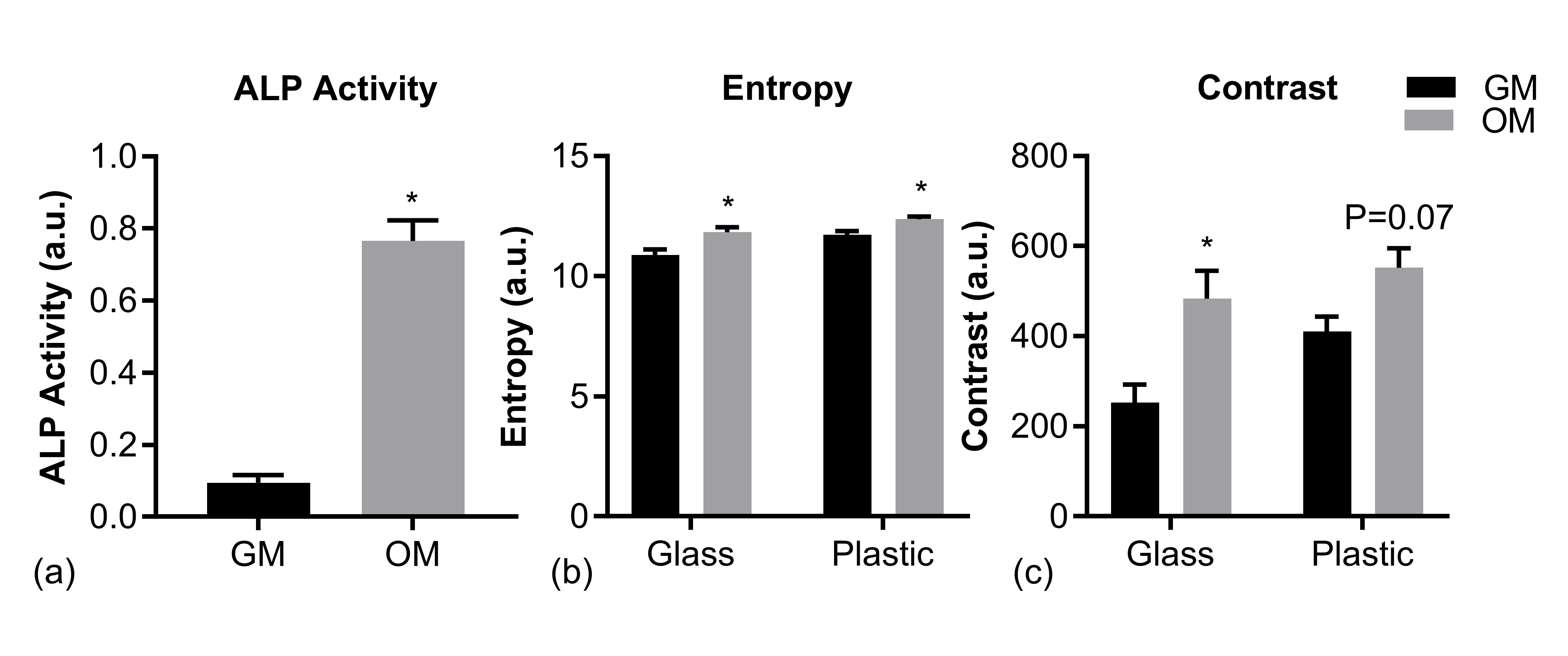

Supplement: Supplemental Material [file TSTA_A_1808432_SM2692.png]
